# Psychopathy Scores Predict Recidivism in High-risk Youth: A Five-year Follow-up Study

**DOI:** 10.1007/s10802-024-01169-x

**Published:** 2024-02-26

**Authors:** Corey H. Allen, Aparna R. Gullapalli, Michaela Milillo, Devin M. Ulrich, Samantha N. Rodriguez, J. Michael Maurer, Eyal Aharoni, Nathaniel E. Anderson, Carla L. Harenski, Gina M. Vincent, Kent A. Kiehl

**Affiliations:** 1 The Mind Research Network, 1101 Yale Blvd NE, Albuquerque, NM 87106-4188 USA; 2 Department of Psychiatry, University of Illinois - Chicago College of Medicine, Chicago, IL 60612 USA; 3 Department of Psychology, University of New Mexico, Albuquerque, NM 87131 USA; 4 Department of Psychology, Georgia State University, P.O. Box 5010, Atlanta, GA 30302-5010 USA; 5 Department of Psychiatry, Law & Psychiatry Program and Implementation Science & Practices Advances Research Center, University of Massachusetts Medical School, 222 Maple Ave, Shrewsbury, MA 01545 USA

**Keywords:** Psychopathy, Juvenile, Delinquency, Impulsivity, Rearrest, Violence

## Abstract

Psychopathic traits have been associated with rearrest in adolescents involved in the criminal legal system. Much of the prior work has focused on White samples, short follow-up windows, and relatively low-risk youth. The current study aimed to evaluate the utility of the Hare Psychopathy Checklist: Youth Version (PCL:YV) for predicting general and violent felony recidivism in a large sample of high-risk, predominantly Hispanic/Latino, male adolescents (*n* = 254) with a five-year follow-up period. Results indicated higher PCL:YV scores and lower full-scale estimated IQ scores were significantly associated with a shorter time to felony and violent felony rearrest. These effects generalized to Hispanic/Latino adolescents (*n* = 193)—a group that faces disproportionate risk of being detained or committed to juvenile correctional facilities in the U.S. These results suggest that expert-rated measures of psychopathic traits and IQ are reliable predictors of subsequent felony and violent felony rearrest among high-risk male adolescents.

**Supplementary Information:**

The online version contains supplementary material available at 10.1007/s10802-024-01169-x.

## Introduction

The construct of psychopathy is characterized by a constellation of traits, including interpersonal and affective deficits, poor decision-making, and impulsivity. The relationship between psychopathic traits and antisocial behaviors, including criminal behavior, has been well-established among adults (Allen et al., 2022; Edwards et al., 2023; Leistico et al., 2008) and adolescents (Braga et al., 2023; Caldwell, 2011; Cauffman et al., 2009; Dolan & Rennie, 2008; Edens & Cahill, 2007; Geerling et al., 2020; Gretton et al., 2004; Hilterman et al., 2014; Khanna et al., 2014; McCuish et al., 2018; Schmidt et al., 2011; Shepherd & Strand, 2016; Sitney et al., 2016; Stockdale et al., 2010; Vincent et al., 2008). Based on this strong relationship between psychopathic traits and antisocial behaviors, it is estimated that crimes stemming from individuals scoring high on psychopathy are responsible for nearly $460 billion in social and economic costs per year, with $56.7 billion being accounted for by juveniles with elevated psychopathic traits (Anderson et al., 1999; Kiehl & Hoffman, 2011). Given the extraordinary social and financial cost of psychopathy to society, there has emerged a heightened interest in identifying individuals scoring high on psychopathic traits during adolescence to predict their likelihood of reoffending over time. Understanding this temporal dimension of antisocial risk carries the potential to identify and disrupt maladaptive and life-course-persistent trajectories.

Though there are a variety of assessments designed to measure psychopathic traits (da Silva et al., 2020; Gillespie et al., 2023), the Hare Psychopathy Checklist-Revised (PCL-R; Hare, 2003) has garnered the most support within the forensic community as a risk assessment tool for predicting violent and general recidivism (Dolan & Doyle, 2000; Khiroya et al., 2009; Walters, 2003). An adolescent version—the Hare Psychopathy Checklist: Youth Version (PCL:YV; Forth & Kosson, 2003)—was later developed to identify youth characterized by elevated psychopathic traits at an early stage in development. Assessment protocols for both the PCL-R and PCL:YV include semi-structured interviews with the participant as well as auxiliary reviews of available participant institutional files (e.g., criminal records). The PCL-R and PCL:YV are commonly used to inform decision-making throughout the criminal legal pipeline—such as sentencing, treatment assessment, and parole—for adults and adolescents, respectively (DeMatteo & Olver, 2022; Salekin et al., 1996).

The predictive utility of the PCL:YV for rearrest has strong empirical support (Caldwell, 2011; Cauffman et al., 2009; Dolan & Rennie, 2008; Edens & Cahill, 2007; Gretton et al., 2004; Hilterman et al., 2014; Khanna et al., 2014; McCuish et al., 2018; Schmidt et al., 2011; Shepherd & Strand, 2016; Sitney et al., 2016; Stockdale et al., 2010; Vincent et al., 2008). These studies report that lifestyle and antisocial psychopathic traits (e.g., impulsivity and early behavioral problems) are most useful in predicting subsequent rearrest. In some samples, affective psychopathic traits (e.g., a lack of empathy) also predict violent rearrest (Braga et al., 2023). However, most studies conducted thus far have focused predominantly on non-Hispanic/Latino samples (see Braga et al., 2023 for a review).

Given the fact that Hispanic youth in the U.S. are at disproportionate risk of being detained or committed to juvenile correctional facilities compared to their White and non-Hispanic/Latino peers (Sickmund et al., 2023), there is an impetus to carefully assess the nature of this relationship across cultures and minorities. While Braga and colleagues’ (2023) results suggest that the percentage of White participants in a study had no significant effect on the relationship between psychopathic traits and offense outcomes, this same result does not specifically suggest the stability of these results within specific ethnic groups (i.e., Hispanic/Latino samples). Studies with more ethnically diverse samples suggest weaker or non-existent relationships between the PCL:YV and antisocial outcomes (Edens & Cahill, 2007). Additionally, though some studies (see Salekin, 2008) were conducted with high portions of Hispanic/Latino participants (47%), analyses were solely conducted on the whole sample rather than constrained to this specific ethnicity. This raises the question of whether previously observed relationships between psychopathic traits and antisocial outcomes would extend to other ethnicities, such as Hispanic/Latino samples.

Another issue is how to characterize the nature of the relationship between psychopathic traits and recidivism risk. Research suggests that the risk associated with psychopathic traits may be mediated by the ability to reason or solve problems, which is commonly operationalized through measures of estimated intelligence (intelligence quotient: IQ). While the majority of the literature suggests a negative relationship between IQ and recidivism (Beaver et al., 2013; Loeber et al., 2012; Lynam et al., 1993; Moffitt et al., 1981; Salekin et al., 2010; Schwartz & Beaver, 2019; Schwartz et al., 2015), IQ has also been shown to interact with psychopathic traits and subsequent outcomes in a variety of ways. Some research suggests that a combination of high IQ and high psychopathic traits increases the risk of reoffending in juveniles (compared to either measure alone) (Hampton et al., 2014), while other research suggests non-interactive negative relationships between IQ and rearrest (Beaver et al., 2013; Loeber et al., 2012; Lynam et al., 1993; Moffitt et al., 1981; Salekin et al., 2010; Schwartz & Beaver, 2019; Schwartz et al., 2015), complex curvilinear relationships (Mears & Cochran, 2013), or no relationship (Salekin et al., 2010) between the measure and outcomes. Thus, the extent to which measurements of psychopathy interact with other constructs, namely, IQ, or predict antisocial outcomes over and beyond other measures comorbid with psychopathic traits (i.e., history of substance dependence [SUD]) in adolescents to predict early adulthood offending and violence in high-risk samples remains unclear.

The current study serves as an important conceptual replication and extension of previous work, evaluating the forensic utility of the PCL:YV, and its underlying factors and facets for assessing risk of general and violent felony rearrest in a predominantly Hispanic/Latino adolescent sample. In addition to assessing risk related to PCL:YV Total scores, we examined a two-factor model of psychopathic traits (Hare, 2003; Harpur et al., 1989; Kennealy et al., 2007). This specific two-factor model of psychopathic traits was established via factor analysis (Harpur et al., 1989; Hare et al., 1990), yielding two correlated overarching factors that held explanatory value for the underlying individual items. Later confirmatory factor analyses suggest each of these factors can be further accounted for by two underlying facets (Vitacco et al., 2005; Kosson et al., 2013). Factor 1 is composed of interpersonal (Facet 1) and affective (Facet 2) facets (e.g., grandiosity and a lack of empathy), and Factor 2 is composed of antisocial (Facet 3) and developmental (Facet 4) facets (e.g., impulsivity and early behavioral problems). Rearrest risk was assessed into early adulthood (i.e., across a five-year follow-up period) while controlling for other variables of interest suggested to be associated with rearrest risk (i.e., IQ and SUD).^1^ Additionally, we tested the potential for interactive effects between these variables (i.e., PCL:YV factor interactions and PCL:YV x IQ interactions) to confer synergistic risk above the variables themselves, as tested in prior literature (Corrado et al., 2004; Hampton et al., 2014; Vincent et al., 2008). The current study serves as one of the largest longitudinal analysis of the relationship between psychopathic traits and rearrest outcomes in a Hispanic/Latino sample. We hypothesized that those who scored higher on the PCL:YV (Braga et al., 2023; Edens et al., 2007; Kennealy et al., 2010; Leistico et al., 2008; Salekin, 2008; Walters, 2003), would be characterized by higher rates of general and violent recidivism, including a shorter time to rearrest for both categories, even when controlling for SUD (Allen et al., 2022; Edwards et al., 2023; Tolou-Shams et al., 2023), and estimated IQ (Schwartz & Beaver, 2019). We hypothesized PCL:YV Total scores, and both Factor 1 and Factor 2 scores to be predictive of felony/violent re-arrest, with Factor 2 scores being more strongly related to the rearrest outcomes compared to Factor 1 scores. Furthermore, while Hispanic/Latino youth are at disproportionate risk of rearrest compared to their White and non-Hispanic/Latino peers, we hypothesize that the aforementioned risk factors will generalize when tested in the Hispanic/Latino subsample.

## Methods

### Participants

The sample consisted of 254 male adolescents from the National Institute of Mental Health (NIMH)–funded SouthWest Advanced Neuroimaging Cohort—Youth (SWANC-Y) sample (R01 MH071896; PI: Kiehl). Participants were assessed during a term of incarceration at a high-risk juvenile correctional facility in New Mexico between June 2007 and March 2011, and had been released for at least 60 months prior to follow-up. Inclusion criteria consisted of fluency in English at or above a fourth-grade reading level and an estimated full-scale IQ over 60 (Ryan & Ward, 1999; Weschler, 2003). The participants ranged in age from 14 to 21 years (*M* = 18.29; *SD* = 1.30) at the time of release from the juvenile correctional facility. Based on National Institutes of Health racial and ethnic classification, 61.0% of the sample self-identified as White, 5.9% as Black/African American, 13.0% as American Indian or Alaskan Native, 20.1% as multiracial/other, and, ethnically, 76.0% as Hispanic/Latino (of which, 66.8% identify as White).^2^

All research protocols were approved by the Ethical and Independent Review Services, the Office for Human Research Protections, and the juvenile correctional facility where data collection occurred (Ethic Committee Name: Salus IRB; Approval Code: 15,050–09). Individuals volunteered to participate after providing written informed consent (if ≥ 18 years of age) or providing written informed assent and parent/guardian written informed consent (if < 18 years of age), which specified consent for follow-up of antisocial outcomes (such as rearrest). They were informed their participation was voluntary and they could stop at any time without penalty. Participants were compensated at a rate consistent with the hourly labor wage at the correctional facilities.

### Assessments and Measures

#### Psychopathy Checklist: Youth Version

The PCL:YV was used to provide a dimensional assessment of psychopathic traits among youth. PCL:YV scores were obtained through a comprehensive review of institutional records and a semi-structured interview that reviews individuals’ school, family, work, and criminal histories, as well as their interpersonal and emotional skills. Interviews were conducted by trained researchers and videotaped for reliability assessment. Individuals were scored on 20 items, scored on a three-point scale from zero (does not apply) to two (definitely applies), based on pervasiveness, severity, and chronicity of each item, for a resulting total score for PCL:YV ranging from zero to 40. Inter-rater-agreement for full scale total scores, calculated from 22 double-rated cases, was high (Cronbach’s Alpha = 0.98). In addition to PCL:YV Total scores, and to provide the most comparable analyses to the adolescent and adult literature, we tested a two-factor and four-facet model (Braga et al., 2023; Hare, 2003; Harpur et al., 1989; Kennealy et al., 2007), with Factor 1 composed of interpersonal (Facet 1) and affective (Facet 2) traits and Factor 2 composed of lifestyle/behavioral (Facet 3) and antisocial/developmental (Facet 4) traits.

#### IQ

Participants’ full-scale IQ was estimated from the Vocabulary and Matrix Reasoning subtests of the Wechsler Adult Intelligence Scale (WAIS-III; Ryan & Ward, 1999; Wechsler, 1997) for those 16 years of age or older and from the Wechsler Intelligence Scale for Children-Fourth Edition (WISC-IV; Sattler & Dumont, 2004; Wechsler, 2003) for those younger than 16 years of age.

#### History of Substance Dependence

Following previous research (Edwards et al., 2023), trained researchers assessed history of substance dependence using the Kiddie Schedule for Affective Disorders and Schizophrenia (K-SADS: Kaufman et al., 1997), summing the total number of substances (alcohol, cannabis, sedatives/hypnotics/anxiolytics, cocaine, opioids, hallucinogens, stimulants, and solvents/inhalants/other) for which an individual met the lifetime dependence diagnostic criteria (*substance dependence* [SUD]; theoretical range: 0 – 8).

#### Recidivism

Recidivism data were compiled using a dataset of criminal case records for the state of New Mexico curated by the Center of Science and Law (www.scilaw.org). The dataset consists of more than 7.27 million records and spans criminal arrests in New Mexico from February 1977 to August 2019 and was obtained via the New Mexico Administrative Office of the Courts (AOC). Each criminal record was parsed into 33 distinct fields, including the individual’s name, date of birth (DOB), last six digits of their social security number (SSN), case number, case type, offense statute, offense date, and offense charge. For each participant, we compiled demographic information (participant’s name, DOB, SSN and release date) from their criminal records. Broad search criteria, including partial matches based on permutations and combinations of the last name, first name, DOB, and SSN from each participant were used to extract rearrest data. Extensive online searches including social media, White Pages, Been Verified, county records, New Mexico AOC website, New Mexico Corrections Department offender search and out of state inmate databases were conducted for the entire sample. This enabled us to compile recidivism data for participants who were not found in the New Mexico AOC database but may have recidivated outside of New Mexico (*n* = 12). These additional broad search criteria also enabled us to identify those that died at any point during (*n* = 8) or following (*n* = 20) the five-year follow-up window in the present study (*n* = 28 in total).

The present study specifically focused on felony and violent felony related recidivism. Felony recidivism was defined as any arrest for a felony offense following the participant’s release-date from the juvenile correctional facility. Felony offenses were further classified into violent felonies, including arrests for assault, battery, robbery, kidnapping, attempted murder, homicide, and related crimes.

In addition to assigning binary outcomes (yes/no) for rearrest, we also recorded time to rearrest as the number of months between discharge from prison and the next arrest or the end of the five-year follow-up window, whichever came first.^3^ For deceased participants who did not reoffend within the specified time-window of five-years (*n* = 8), their time at risk was calculated as the number of days between their release date and date of death.

### Statistical Analyses

The two main recidivism categories of interest were felony arrest and violent felony arrest. Time to rearrest for each of these categories was visualized with Kaplan-Meier survival curves. Cox proportional-hazard regression was employed to test the predictive utility of the PCL:YV (Total, Factor scores, Factor interactions [Corrado et al., 2004; Vincent et al., 2008] and Facets), IQ, and their interactions (i.e., PCL:YV Total, Factors, and Facets x IQ). Additional risk factors noted in the literature (Aharoni et al., 2013; Kiehl et al., 2018; Ray & Richardson, 2017; Schwartz, 2021; Tolou-Shams et al., 2023) - age at release, number of SUDs, and traumatic brain injury (TBI), were also examined (Cappelle et al., 2023; Olson & Vaughn, 2023). Cox proportional-hazard regression results in relative risk-ratios, which convey the additional percentage risk of an outcome per single value added to the independent variables included in the model. All variables of interest were first tested for their bivariate predictive utility for each outcome. These results later informed multivariate models for each outcome category, allowing identification of rearrest related variables to be covaried in multivariate models in order to establish the predictive utility of psychopathic traits and their interactions with IQ while holding other rearrest related variables constant. Variables that were not found to be predictive of rearrest in bivariate analyses were not included in further multivariate analyses. Thus, the multivariate models took the following structure: Block (1) IQ and SUD; Block (2) PCL:YV Measures (Total, Factor, and Facet Scores tested in separate models), and Block (3)^4^ interaction terms between psychopathic traits and IQ. All variables in the multivariate models were mean-centered. Cox regressions were performed using the survival package in *RStudio* (RStudio, 2020; Theneau, 2020), and all primary analyses were conducted in the full sample (*n* = 254), and also in a constrained sub-sample of those that self-reported as Hispanic/Latino (*n* = 193).

## Results

### Descriptive Statistics

#### Psychopathic Traits, IQ, SUD, & TBI

An analysis of all PCL:YV items indicated high internal reliability (Cronbach's alpha = 0.83). PCL:YV Total scores ranged from 2 to 35 (*M* = 23.35; *SD* = 6.10), Factor 1 (interpersonal/affective) scores from 0 to 15 (*M* = 6.64; *SD* = 3.01), and Factor 2 (lifestyle/antisocial) scores from 1 to 20 (*M* = 14.62; *SD* = 3.29). As expected, PCL:YV factor scores were positively correlated with each other, *r*(252) = 0.54, *p* < 0.001 (see Table 2). Compared to other forensic youth samples (considering both weighted means presented in meta-analyses, as well as other larger forensic sample analyses focusing on the PCL:YV), the present sample is characterized by higher PCL:YV Total scores (see Cauffman et al., 2009; Edens et al., 2007; Vincent et al., 2008).

The mean full-scale IQ estimate in this sample was 92.15 (*SD* = 12.14: see Table 1). As expected, IQ was negatively correlated with PCL:YV Factor 2 scores *r*(252) = -0.19, *p* = 0.002 (see Table 2). 86.6% of the sample met criteria for at least one SUD, and 61.4% of the sample met criteria for TBI. The mean number of SUDs in this sample was 2.23 (*SD* = 1.55: see Table 1). As expected, SUD was positively correlated with youth psychopathic traits (*p*’s < 0.005).

**Table 1:** Participant demographics and assessment scores

	**Mean**	***SD***	**Min.**	**Max.**	***n***	**Overall sample (%)**
Age (years)	18.29	1.30	14.35	21.02		
IQ	92.15	12.14	63	140		
PCL:YV Total	23.53	6.10	2	35		
PCL:YV Factor 1	6.64	3.01	0	15		
PCL:YV Factor 2	14.62	3.29	1	20		
PCL:YV Facet 1	2.19	1.82	0	7		
PCL:YV Facet 2	4.46	1.79	0	8		
PCL:YV Facet 3	6.39	2.03	0	10		
PCL:YV Facet 4	8.25	1.69	0	10		
SUD	2.23	1.55	0	8	220	86.6
TBI					156	61.4

Age corresponds to participant age at their release date. For the purposes of this table, SUD is presented both linearly and dichotomously (representing the percentage of participants that meet criteria for at least one substance use dependency)

**Table 2:** Pearson Correlation Matrix

	1	2	3	4	5	6	7	8	9
1. Age	—								
2. IQ	0.09	—							
3. PCL:YV Factor 1	0.06	0.03	—						
4. PCL:YV Factor 2	-0.07	-0.19**	0.54***	—					
5. PCL:YV Total	-0.01	-0.10	0.85***	0.88***	—				
6. SUD	0.09	0.05	0.22**	0.30***	0.31***	—			
7. PCL:YV Facet 1	0.08	0.13*	0.84***	0.34***	0.65***	0.16*	—		
8. PCL:YV Facet 2	0.01	-0.08	0.84***	0.56***	0.78***	0.19**	0.41***	——	
9. PCL:YV Facet 3	-0.04	-0.13*	0.48***	0.90***	0.80***	0.28***	0.34***	0.47***	—
10. PCL:YV Facet 4	-0.09	-0.22***	0.45***	0.85***	0.75***	0.23***	0.25***	0.51***	0.54***

*p < 0.05; **p < 0.01; ***p < 0.001

#### Recidivism Outcomes

**Fig. 1:**
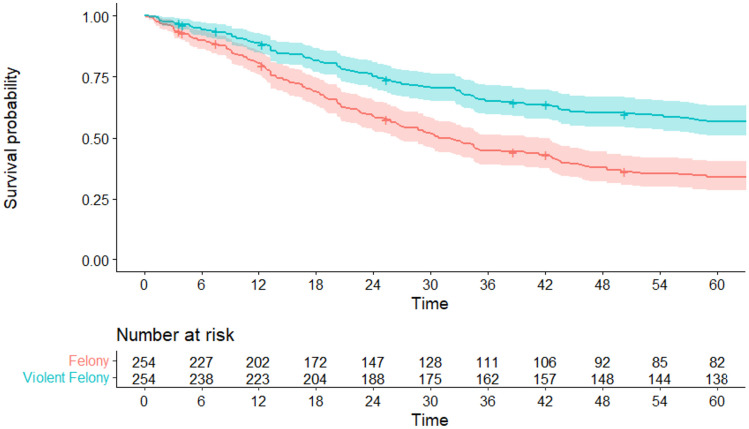
Kaplan-Meier curves showing rearrest rates (in months) with 95% confidence intervals and survival risk tables across Felony Rearrest and Violent Felony Rearrest

Of the 254 juvenile males in this study, 164 (~65% of the sample) were rearrested for a felony offense in the five-year follow up period (see Fig. 1).^5^ The time to arrest from release date ranged from 3 days to 58.88 months (*M* = 21.34 months, *SD* = 14.37 months). Of those arrested for a felony, 108 (~43% of the sample) of those were arrested for violent felonies (see Fig. 1).^6^ The time to violent felony arrest ranged from 13 days to 58.88 months (*M* = 23.49 months, *SD* = 15.39 months).

### Recidivism Predictive Analyses

#### Bivariate Analyses: Full Sample

Bivariate Cox proportional-hazard regressions were performed across the two categories of arrests to assess the individual predictive utility of the variables of interest and to inform variable selection for multivariate models.

**Fig. 2:**
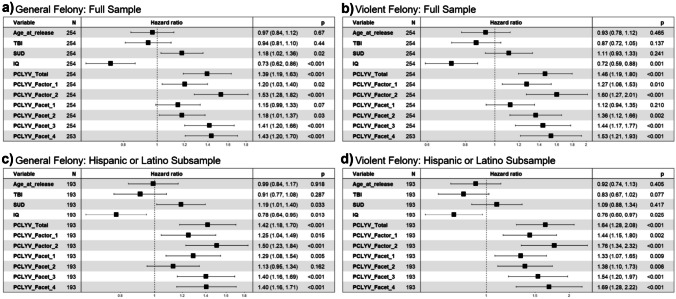
Centered and scaled results of Cox proportional-hazard regression bivariate analyses examining the predictive effect of age at release, TBI, SUD, IQ, PCL:YV Total, Factor 1, Factor 2, Facet 1, Facet 2, Facet 3, and Facet 4 on **a** general felony and **b** violent felony rearrest for the full sample, and **c** general felony and **d** violent felony rearrest for the Hispanic/Latino subsample. Scaling includes normalization of values via the mean and standard deviation: analogous to a z-score. Sample size, Beta HR (95% CI for HR), and p-values are shown for each measure

As predicted, higher psychopathic traits (i.e., PCL:YV Total, Factor 1, Factor 2, Facet 1, Facet 2, Facet 3, and Facet 4 scores), lower IQ, and higher number of SUDs were each significantly associated with time to felony rearrest (*p* < 0.001, *p* = 0.017, *p* < 0.001, *p* = 0.066 [one-tailed], *p* = 0.033, *p* < 0.001, *p* < 0.001 *p* < 0.001, & *p* = 0.021 respectively; see Fig. 2a). Age at release and TBI history were not significant predictors of time to general felony rearrest and therefore, were not included as covariates in subsequent analyses (*p*’s > 0.05: see Fig. 2a).

As predicted, higher psychopathic traits (i.e., PCL:YV Total, Factor 1, Factor 2, Facet 2, Facet 3, and Facet 4) and lower IQ were each significantly associated with time to violent felony rearrest (*p* < 0.001, *p* = 0.010, *p* < 0.001, *p* = 0.002, *p* < 0.001, *p* < 0.001, & *p* = 0.001, respectively; see Fig. 2b). PCL:YV Facet 1, age at release, SUD, and TBI history were not significant predictors of time to violent felony rearrest (*p*’s > 0.05: see Fig. 2b).

#### Bivariate Analyses: Hispanic/Latino Sub-Sample

Identical procedures outlined in "Bivariate Analyses: Full Sample" section were applied to the sub-sample participants identifying as Hispanic/Latino. Importantly, all primary bivariate effects of SUD, IQ, and psychopathic traits remained significant in this subset of participants(*n* = 193) as reported in the overall larger sample of participants (see Fig. 2c, d), suggesting robust effects of the relationship between these measures of interest and antisocial outcomes specifically for Hispanic/Latino youth.^7^

For general felony rearrest (126 events), and as predicted, higher psychopathic traits (i.e., PCL:YV Total, Factor 1, Factor 2, Facet 1, Facet 3, and Facet 4), lower IQ, and higher number of SUDs were each significantly associated with time to felony rearrest (*p* < 0.001, *p* = 0.015, *p* < 0.001, *p* = 0.005, *p* < 0.001, *p* < 0.001, *p* = 0.013, & *p* = 0.033 respectively; see Fig. 2c). Age at release, TBI, and Facet 2 were not significant predictors of time to general felony rearrest (*p*’s > 0.05: see Fig. 2c).

For violent felony rearrest (82 events), and as predicted, higher psychopathic traits (i.e., PCL:YV Total, Factor 1, Factor 2, Facet 1, Facet 2, Facet 3, and Facet 4) and lower IQ were each significantly associated with time to violent felony rearrest (*p* < 0.001, *p* = 0.002, *p* < 0.001, *p* = 0.009, *p* = 0.006, *p* < 0.001, *p* < 0.001, & *p* = 0.025, respectively). Age at release, SUD, and TBI were not significant predictors of time to violent felony rearrest (*p*’s > 0.05: see Fig. 2d).

#### Multivariate Analyses: Full Sample

Multivariate Cox proportional-hazard regressions were performed across the two categories of arrests to assess the predictive utility of psychopathic traits while holding constant other variables of interest. More specifically, a blockwise multivariate method was used to test 1) whether psychopathic traits predict the arrest outcomes while controlling for other variables of interest (i.e., SUD & IQ),^8^ and 2) whether interaction effects add incremental utility to these models. Thus, the multivariate analyses for each outcome category included: Block 1) SUD & IQ, Block 2) PCL:YV measures (Total, Factor, and Facet Scores tested in separate models), and Block 3) PCL:YV and IQ interaction terms.

**Fig. 3:**
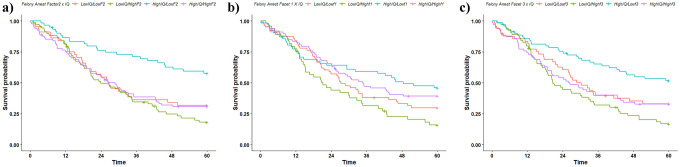
Kaplan-Meier curves showing felony rearrest rates (in months) based on median splits of IQ and **a** PCL:YV Factor 2, **b** PCL:YV Facet 1, and **c** PCL:YV Facet 3 for the sake of visualizing observed significant interaction effects

For general felony rearrest, a significant overall effect was found for Block 1 (*p* < 0.001). Lower IQ and higher SUDs scores were each significantly associated with time to felony rearrest (*p* < 0.001 & *p* < 0.05, respectively). As predicted, Block 2 yielded a significant improvement for Total, Factor, and Facet models for felony rearrest (*p’s* < 0.01). PCL:YV Total and Factor 2 (and accordingly, Facets 3 and 4) scores remained significantly positively related to felony rearrest in the multivariate model (*p’s* < 0.05). Factor 1 (and Facets 1 and 2) scores were not significant. Notably, effects of SUD on general felony rearrest become non-significant when psychopathic traits were included in the model. Block 3 did not significantly improve the variance accounted for in either Total or Factor models for felony rearrest (*p’s* > 0.10), yet showed moderate improvement in the Facet model. This suggests that, overall, the variance accounted for by the interactions is not greater than the variance accounted for by the individual factors. Although Block 3 did not significantly improve Total or Factor models for felony rearrest, and only moderately improved the Facet model for rearrest, Factor 2 × IQ, Facet 1 × IQ, and Facet 3 × IQ emerged as significant predictors in the full multivariate models (*p’s* < 0.05: see Table 3; see Fig. 3 for a visualization of the interaction effects). A consistent interaction interpretation is observed across the three PCL:YV measures in conjunction with IQ: high IQ is a protective factor for felony rearrest, except in the case of accompanying high psychopathic traits (be it interpersonal, impulsive, or antisocial traits), whereas low IQ generally exacerbates the rearrest risk afforded by high psychopathic traits.

**Table 3:** Effect of PCL:YV Total and Factor score blockwise multivariate models on felony and violent felony rearrest in full sample

		**Block 1**		**Block 2**		**Block 3**			
**Model**	**Predictor**	**coef(SE)**	**exp(coef)**	**coef(SE)**	**exp(coef)**	**coef(SE)**	**exp(coef)**	**R**^**2**^	**ΔR**^**2**^
**Felony (Total)**									
Block 1	SUD	0.11(.05)	1.12*	0.07(.05)	1.07	0.06(.05)	1.07		
	IQ	-0.03(0.01)	0.97***	-0.02(.01)	0.98***	-0.03(0.01)	0.97***	18.82***	
Block 2	PCL:YV Total			0.05(.01)	1.05**	0.05(0.01)	1.05**	28.21***	11.18**
Block 3	PCL:YV Total x IQ					0.00(0.00)	1.00	28.52***	1.17
**Felony (Factor)**									
Block 1	SUD	0.11(.05)	1.12*	0.06(.05)	1.06	0.06(0.05)	1.06		
	IQ	-0.03(0.01)	0.97***	-0.02(.01)	0.98**	-0.02(0.01)	0.98**	18.72***	
Block 2	PCL:YV Factor 1			0.00(0.03)	1.00	0.01(0.04)	1.01		
	PCL:YV Factor 2			0.11(0.04)	1.11**	0.10(0.04)	1.11**	29.90***	15.25***
Block 3	PCL:YV F1 x F2					0.00(0.01)	1.00		
	PCL:YV Factor 1 × IQ					0.00(0.00)	1.00		
	PCL:YV Factor 2 × IQ					0.01(0.00)	1.01*	30.77***	5.31
**Felony (Facet)**									
Block 1	SUD	0.11(0.05)	1.12*	0.05(0.05)	1.06	0.06(0.05)	1.06		
	IQ	-0.03(0.01)	0.97***	-0.02(0.01)	0.98**	-0.02(0.01)	0.98**	18.16***	
Block 2	PCL:YV Facet 1			0.05(0.05)	1.06	0.05(0.05)	1.05		
	PCL:YV Facet 2			-0.06(0.06)	0.94	-0.05(0.06)	0.95		
	PCL:YV Facet 3			0.09(0.05)	1.10^a^	0.11(0.06)	1.11^a^		
	PCL:YV Facet 4			0.15(0.07)	1.16*	0.12(0.07)	1.13^a^	30.85***	16.57**
Block 3	PCL:YV Facet 1 × IQ					-0.01(0.00)	0.99*		
	PCL:YV Facet 2 × IQ					0.00(0.01)	1.00		
	PCL:YV Facet 3 × IQ					0.01(0.01)	1.01*		
	PCL:YV Facet 4 × IQ					0.00(0.01)	1.00	36.44***	7.79^a^
**Violent Felony (Total)**									
Block 1	SUD	0.08(0.06)	1.08	0.02(0.07)	1.02	0.01(0.07)	1.10		
	IQ	-0.03(0.01)	0.97**	-0.03(0.01)	0.97**	-0.03(0.01)	0.97**	11.85**	
Block 2	PCL:YV Total			0.06(0.02)	1.06**	0.06(0.02)	1.06**	21.97***	11.54**
Block 3	PCL:YV Total x IQ					0.00(0.00)	1.00	23.55***	0.02
**Violent Felony (Factor)**									
Block 1	SUD	0.08(0.06)	1.08	0.01(0.07)	1.01	0.00(0.07)	1.00		
	IQ	-0.03(0.01)	0.97**	-0.03(0.01)	0.98**	-0.03(0.01)	0.98**	11.85**	
Block 2	PCL:YV Factor 1			0.02(0.04)	1.02	0.01(0.04)	1.01		
	PCL:YV Factor 2			0.12(0.04)	1.13**	0.13(0.05)	1.14**	23.56***	14.98**
Block 3	PCL:YV F1 x F2					0.01(0.01)	1.01		
	PCL:YV Factor 1 × IQ					0.00(0.00)	1.00		
	PCL:YV Factor 2 × IQ					0.00(0.00)	1.00	28.55***	0.76
**Violent Felony (Facet)**									
Block 1	SUD	0.07(0.06)	1.07	0.01(0.07)	1.01	0.02(0.07)	1.11		
	IQ	-0.03(0.01)	0.97**	-0.02(0.01)	0.98**	-0.03(0.01)	0.97**	11.61**	
Block 2	PCL:YV Facet 1			0.00(0.06)	1.00	-0.01(0.06)	0.99		
	PCL:YV Facet 2			0.06(0.07)	1.06	0.07(0.07)	1.07		
	PCL:YV Facet 3			0.11(0.06)	1.11	0.08(0.07)	1.08		
	PCL:YV Facet 4			0.14(0.09)	1.14	0.16(0.09)	1.17^a^	23.33**	15.01**
Block 3	PCL:YV Facet 1 × IQ					0.00(0.01)	1.00		
	PCL:YV Facet 2 × IQ					0.00(0.01)	1.00		
	PCL:YV Facet 3 × IQ					-0.01(0.01)	0.99		
	PCL:YV Facet 4 × IQ					0.01(0.01)	1.01	27.72**	2.36

Results of blockwise multivariate Cox regression analyses examining the predictive effect of Total, Factor, and Facet models on felony, and Total, Factor, and Facet models on violent felony. All variables were mean centered. Table reports unstandardized B (coef), standard error, relative risk ratio (exp[coef]), chi-square, and change in chi-square per block of each model

*p < 0.05; **p < 0.01; ***p < 0.001 (two-tailed)

^a^*p* < 0.05 (one-tailed, a priori)

Similarly, for violent felony rearrest, a significant overall effect was found for Block 1 (*p* < 0.01). Lower IQ was significantly associated with time to violent felony rearrest (*p* < 0.001), while SUD was not a significant predictor. As predicted, Block 2 yielded a significant improvement for Total, Factor, and Facet models for violent felony rearrest (*p’s* < 0.01), with PCL:YV Total and Factor 2 scores remaining significantly positively associated with violent felony rearrest in the multivariate model (*p’s* < 0.01), while Factor 1 (as well as Facet 1, 2, 3, and 4) scores were not significant. Block 3 did not significantly improve the variance accounted for in either Total, Factor, or Facet models for violent felony rearrest (*p’s* > 0.10: see Table 3), suggesting that the variance accounted for by interactions is not greater than that accounted for by the individual factors.

In summary, as hypothesized, our results suggest that psychopathic traits were reliable predictors for general and violent felony rearrest, even while controlling for other variables of interest (i.e., SUDs & IQ), and interactions between psychopathic traits and IQ confer limited additional variance accounted for in the case of general felony rearrest.

#### Multivariate Analyses: Hispanic/Latino Sub-Sample

Identical procedures outlined in "Multivariate Analyses: Full Sample" section were applied to the Hispanic/Latino sub-sample (*n* = 193). A significant overall effect was found for Block 1 (*p* < 0.01). Lower IQ and higher SUDs scores were each significantly associated with time to felony rearrest (*p’s* < 0.05). As predicted, Block 2 yielded a significant improvement for both Total, Factor, and Facet models for felony rearrest (*p’s* < 0.01), with PCL:YV Total, Factor 2, Facet 1, and Facet 4 scores remaining significantly positively related to felony rearrest in the multivariate model (*p’s* < 0.05), while Factor 1, Facet 2, and Facet 3 scores were not significant. Notably, effects of SUD on general felony rearrest become non-significant when psychopathic traits were included in the model. Block 3 did not significantly improve the variance accounted for in either Total, Factor, or Facet models for felony rearrest (*p’s* > 0.10), suggesting that the variance accounted for by the interactions is not greater than the variance accounted for by the individual factors.

Similarly, for violent felony rearrest, a significant overall trend of a one-tailed effect was found for Block 1 (*p* < 0.10). Lower IQ was significantly associated with time to violent felony rearrest (*p* < 0.05), while SUD was not a significant predictor. As predicted, Block 2 yielded a significant improvement for Total, Factor, and Facet models for violent felony rearrest (*p’s* < 0.01). PCL:YV Total, Factor 2, and Facet 4 scores remaining significantly positively associated with violent felony rearrest in the multivariate model (*p’s* < 0.05). Factor 1, and Facets 1, 2, and 3 scores were not significant. Block 3 did not significantly improve the variance accounted for in either Total or Factor models for violent felony rearrest (*p’s* > 0.10: see Table 4), suggesting that the variance accounted for by interactions is not greater than that accounted for by the individual factors.

**Table 4:** Effect of PCL:YV Total and Factor score blockwise multivariate models on felony and violent felony rearrest in Hispanic/Latino sub-sample

		**Block 1**		**Block 2**		**Block 3**			
**Model**	**Predictor**	**coef(SE)**	**exp(coef)**	**coef(SE)**	**exp(coef)**	**coef(SE)**	**exp(coef)**	**R**^**2**^	**ΔR**^**2**^
**Felony (Total)**									
Block 1	SUD	0.12(0.05)	1.13*	0.07(0.06)	1.07	0.06(0.06)	1.07		
	IQ	-0.02(0.01)	0.98*	-0.02(0.01)	0.98*	-0.02(0.01)	0.98*	10.79**	
Block 2	PCL:YV Total			0.05(0.02)	1.05**	0.05(0.02)	1.05**	19.45***	9.69**
Block 3	PCL:YV Total x IQ					0.00(0.00)	1.00	19.53**	0.57
**Felony (Factor)**									
Block 1	SUD	0.12(.05)	1.13*	0.06(0.06)	1.06	0.06(0.06)	1.06		
	IQ	-0.02(0.01)	0.98*	-0.02(0.01)	0.98*	-0.02(0.01)	0.98*	10.79**	
Block 2	PCL:YV Factor 1			0.01(0.04)	1.01	0.02(0.04)	1.02		
	PCL:YV Factor 2			0.10(0.04)	1.11*	0.10(0.04)	1.11*	20.10***	11.62**
Block 3	PCL:YV F1 x F2					0.00(0.01)	1.00		
	PCL:YV Factor 1 × IQ					0.00(0.00)	1.00		
	PCL:YV Factor 2 × IQ					0.00(0.00)	1.00	20.73**	0.37
**Felony (Facet)**									
Block 1	SUD	0.12(0.05)	1.13*	0.06(0.06)	1.06	0.06(0.06)	1.06		
	IQ	-0.02(0.01)	0.98*	-0.22(0.01)	0.98*	-0.02(0.01)	0.98^a^	10.79**	
Block 2	PCL:YV Facet 1			0.13(0.06)	1.14*	0.12(0.06)	1.13*		
	PCL:YV Facet 2			-0.12(0.06)	0.89	-0.11(0.07)	0.90		
	PCL:YV Facet 3			0.09(0.06)	1.10	0.11(0.06)	1.11^a^		
	PCL:YV Facet 4			0.16(0.08)	1.18*	0.15(0.08)	1.12*	27.61***	18.49**
Block 3	PCL:YV Facet 1 × IQ					-0.01(0.01)	0.99		
	PCL:YV Facet 2 × IQ					0.01(0.01)	1.01		
	PCL:YV Facet 3 × IQ					0.01(0.01)	1.01		
	PCL:YV Facet 4 × IQ					-0.01(0.01)	0.99	33.24***	3.15
**Violent Felony (Total)**									
Block 1	SUD	0.06(0.07)	1.06	-0.03(0.08)	0.97	-0.03(0.08)	0.97		
	IQ	-0.02(0.01)	0.98*	-0.02(0.01)	0.98*	-0.02(0.01)	0.98^a^	5.68^a^	
Block 2	PCL:YV Total			0.08(0.02)	1.09***	0.08(0.02)	1.09***	20.48***	15.97***
Block 3	PCL:YV Total x IQ					0.00(0.00)	1.00	21.84***	0.00
**Violent Felony (Factor)**									
Block 1	SUD	0.06(0.07)	1.06	-0.03(0.08)	0.97	-0.04(0.08)	0.91		
	IQ	-0.02(0.01)	0.98*	-0.02(0.01)	0.98*	-0.02(0.01)	0.98^a^	5.68^a^	
Block 2	PCL:YV Factor 1			0.05(0.05)	1.05	0.03(0.05)	1.03		
	PCL:YV Factor 2			0.15(0.05)	1.16**	0.16(0.05)	1.17**	21.82***	18.51**
Block 3	PCL:YV F1 x F2					0.01(0.01)	1.01		
	PCL:YV Factor 1 × IQ					0.00(0.00)	1.00		
	PCL:YV Factor 2 × IQ					0.00(0.00)	1.00	27.66***	1.35
**Violent Felony (Facet)**									
Block 1	SUD	0.06(0.07)	1.06	-0.04(0.08)	0.96	-0.03(0.08)	0.97		
	IQ	-0.02(0.01)	0.98*	-0.02(0.01)	0.98*	-0.02(0.01)	0.98^a^	5.68^a^	
Block 2	PCL:YV Facet 1			0.09(0.07)	1.10	0.08(0.07)	1.08		
	PCL:YV Facet 2			-0.01(0.08)	0.99	0.00(0.09)	1.00		
	PCL:YV Facet 3			0.11(0.08)	1.12	0.09(0.08)	1.10		
	PCL:YV Facet 4			0.22(0.10)	1.25*	0.24(0.10)	1.23*	23.35**	19.89**
Block 3	PCL:YV Facet 1 × IQ					0.00(0.01)	1.00		
	PCL:YV Facet 2 × IQ					0.01(0.01)	1.01		
	PCL:YV Facet 3 × IQ					-0.01(0.01)	0.99		
	PCL:YV Facet 4 × IQ					0.00(0.01)	1.00	27.49**	2.02

Results of blockwise multivariate Cox regression analyses examining the predictive effect of Total, Factor, and Facet models on felony, and Total, Factor, and Facet models on violent felony. All variables were mean centered. Table reports unstandardized B (coef), standard error, relative risk ratio (exp[coef]), chi-square, and change in chi-square per block of each model

*p < 0.05; **p < 0.01; ***p < 0.001 (two-tailed)

^a^*p* < 0.05 (one-tailed, a priori)

Importantly, all primary multivariate effects suggesting that psychopathic traits were reliable predictors for general and violent felony rearrest. These results were robust to controlling for other variables of interest (i.e., SUDs & IQ) and the results also generalized to the Hispanic/Latino subset (*n* = 193) of the larger sample (see Table 4).

## Discussion

The purpose of this study was to assess the forensic utility of the PCL:YV for predicting general and violent recidivism in a large sample of predominantly Hispanic/Latino high-risk male adolescents up to five years post-release. Analyses controlled for additional variables of interest (i.e., IQ and SUD), and also considered the interactive effects of psychopathic traits and IQ. As hypothesized, and consistent with prior literature, we observed that higher juvenile psychopathic traits, lower estimated IQ, and meeting criteria for a higher number of substance dependencies (at a bivariate level), were predictive of shorter time to and higher rates of rearrest into adulthood. Psychopathic traits were the strongest predictor (see Fig. 2), even while controlling for the aforementioned measures (see Tables 3 and 4). These effects generalized strongly to participants identifying as Hispanic/Latino (see "Bivariate Analyses: Hispanic/Latino Sub-Sample", "Multivariate Analyses: Hispanic/Latino Sub-Sample" sections). This study illustrates the utility and generalizability of the PCL:YV in a high-risk sample.

PCL:YV Factor 1 and Factor 2 scores were associated with both felony and violent felony rearrest outcomes. Although PCL:YV Factor 2, and its facets (Facets 3 and 4)—measuring specifically lifestyle/antisocial psychopathic traits, respectively—demonstrated the strongest predictive effects for both outcome categories, it is important to note that PCL:YV Total scores, a summary metric most commonly used for risk assessment, showed comparable effects, largely consistent with previous findings (Braga et al., 2023; Caldwell, 2011; Cauffman et al., 2009; Dolan & Rennie, 2008; Edens & Cahill, 2007; Gretton et al., 2004; Hilterman et al., 2014; Khanna et al., 2014; McCuish et al., 2018; Schmidt et al., 2011; Shepherd & Strand, 2016; Sitney et al., 2016; Stockdale et al., 2010; Vincent et al., 2008: see Tables 3 and 4). The predictive effects of psychopathic traits on felony rearrest persisted even while controlling for the number of SUDs, a variable comorbid with psychopathy (Hemphill et al., 1994). Models including all Facet measures (compared to Total scores or Factor scores) largely performed the best in predicting subsequent rearrest (as assessed by R^2^). The robustness of these effects (i.e., the strong positive relationship between psychopathic traits and felony and violent felony rearrest) carries importance not only for risk assessments aimed at prediction, but also risk assessments focused on amenability to treatment.

While psychopathy has traditionally been viewed as a “treatment-resistant” cluster of personality traits, emerging research suggests that interventions tailored to individual traits are more successful in curbing antisocial behaviors as compared to non-tailored treatments—a concept commonly referred to as risk-needs-responsivity (Anderson & Kiehl, 2014; Andrews et al., 1990a, b; Simourd & Hoge, 2000; Taxman & Smith, 2021). Indeed, research suggests that adolescents scoring high on the PCL:YV who received trait-tailored treatment—instead of receiving “treatment as usual”—were nearly three times less likely to violently reoffend upon release than those who received standard treatment (Caldwell et al., 2006). A necessary prerequisite to trait-tailored interventions, though, is a comprehensive and accurate assessment of treatment relevant traits (i.e., psychopathic traits). While our effects suggest the importance of antisocial developmental behavioral patterns for the prediction of outcomes, we also find interpersonal/affective traits (i.e., Factor 1 and Facet 2: see Fig. 3) may also have utility in predicting *specific* outcomes, such as rearrest for violent felony offenses. While these effects were limited to bivariate analyses, they are largely in line with effects observed in prior literature (Braga et al., 2023). Additionally, literature suggests that assessing interpersonal/affective traits has added practical utility when considering treatment amenability (Caldwell et al., 2006; Caldwell et al., 2011), strengthening the arguments behind careful and complete administration of the PCL:YV for its use in the criminal legal services.

The present results contribute to the literature about the generalizability of the PCL:YV. The PCL:YV has emerged as a robust estimate of psychopathic traits, and it has strong predictive utility. Here we found that the PCL:YV generalized to Hispanic youth from a very high-risk adolescent sample. Hispanic/Latino youth are more likely to be detained or committed to juvenile correctional facilities than their White and non-Hispanic/Latino peers (Sickmund et al., 2023), leaving them at disproportionate risk of being at the receiving end of criminal legal decisions. For this very same reason, Hispanic/Latino youth can also disproportionately benefit from accurate assessments of risks in cases of mitigating circumstances, and the same case can be made regarding risk-needs-responsivity assessments. But, importantly, these potentially beneficial instances rely on the strength of relationships between traits of interest (e.g., psychopathic traits) and outcomes of interest (e.g., risk of reoffense), for which our present analyses provide novel support.

Our results also observed a negative relationship between estimated IQ and felony and violent felony rearrest, largely consistent with prior literature (Beaver et al., 2013; Loeber et al., 2012; Lynam et al., 1993; Moffitt et al., 1981; Schwartz et al., 2015; Schwartz & Beaver, 2019, though see: Salekin et al., 2010). There are multiple possible interpretations of these effects. On one hand, intellectual functioning may play a protective role against risky decision making. On the other hand, it could decrease individuals’ likelihood of being *caught* in risky situations, an explanation supported by previous findings of positive associations between IQ and criminality more generally (Schwartz & Beaver, 2019).

While psychopathic traits and estimated IQ demonstrated positive and negative associations, respectively, between felony and violent felony rearrest, we note that interactions of the two measures also emerged as significant predictors for felony rearrest in the full sample while controlling for each measure individually. In the literature, theory derived from Cleckley (1941) suggests that those high in both measures demonstrate an elevated risk for subsequent antisocial behavior (Hampton et al., 2014; Munoz et al., 2008), specifically noting that if an individual is both intelligent and conniving or manipulative (i.e., a high PCL:YV Factor 1 × IQ interaction term) one may expect that individual to be immersed more fully in self-serving antisocial behaviors. Notably, our effects do not completely mirror this theory. Instead we observe that a lower IQ exacerbates the rearrest risk afforded by psychopathic traits (see Fig. 3 for a visualization of this interaction effect for Felony Rearrest across PCL:YV Factor 2, Facet 1, and Facet 3 traits). Thus, it may be the case that high psychopathic traits, in combination with low IQ may denote a special rearrest risk that is not captured by either measure individually. That said, it is important to note that the independent effects of the individual factors (e.g., PCL:YV Factor 2 and IQ) are much stronger predictors, suggesting that the parts may be more important than their interactions when considering risk of future rearrest. Likewise, though the effect was significant for the interaction terms themselves (albeit a small effect sizes), the steps including the interaction in the multivariate Total and Factor models did not significantly improve the R^2^ of the multivariate regression, and the interaction step in the Facet model only moderately improved the variance accounted for, reducing any strong conclusions to be drawn from the effect. Accordingly, these effects were also not observed in the Hispanic/Latino subsample. Regardless, these results potentially suggest the value of a more nuanced view regarding the relationships between IQ, psychopathy, and antisocial outcomes, one that considers not only linear relationships between the measures and outcomes, but also considers whether there are certain combinatorial subsets of the measures that may confer additional risk outside of the linear relationships.

### Study Limitations and Future Directions

A number of limitations must be considered alongside the results presented. Although the effects presented above apply to the present sample of high-risk adolescent boys, it is unclear whether the results would generalize to a similar sample of high-risk adolescent girls (Vincent et al., 2008). Likewise, while the presence of TBI has been suggested as a risk factor for subsequent rearrest in the literature (e.g., Ray & Richardson, 2017; Schwartz, 2021), we did not observe such effects (see Table 3: Fig. 2); these effects might be most prevalent in older samples rather than in adolescents, or could be explained by the relatively low base rates of TBI in other samples compared to the present one (Ray & Richardson, 2017). Finally, while our results provide support to the idea that the PCL:YV may act as a risk assessment tool for future recidivism, we did not directly collect or compare its performance to other assessments (e.g., The Structured Assessment of Violence Risk in Youth; Borum et al., 2003), limiting the conclusions that can be drawn regarding the convergent or discriminant validity of this scale. A similar limitation of our results is that they are restricted to examining recidivism for those who were apprehended based on official records. While these results can speak to those most likely to be apprehended, they do not directly speak to those who may be committing crime yet not getting caught. Thus, future longitudinal work aimed at further understanding additional risk and protective factors in adolescence that contribute to criminal behavior and violence in adulthood is necessary to inform risk assessment, prevention, and treatment efforts in this population, and other demographically diverse populations.

### Conclusions

Consistent with our hypotheses, our prospective five-year follow-up study suggests that higher PCL:YV scores (specifically, higher Total, Factor, and Facet scores) were associated with a shorter time to and higher rates of felony and violent felony rearrest, even when controlling for other significant predictors (such as lower IQ and more SUDs). Additionally, these effects generalize to a Hispanic/Latino subsample. Mental health professionals conducting assessments for the courts are often required to make recommendations regarding the risk for future offending, and specifically, violent offending, as these decisions can have life-changing implications for adolescents involved in the legal system. Thus, it is imperative to have accurate, empirically supported risk-assessment tools to inform triage and treatment decisions regarding youth involved in the criminal legal system. These findings contribute to the existing literature suggesting that psychopathic traits and intellectual functioning in adolescence are important predictors of repeat offending and violence into early adulthood, and support the use of the PCL:YV as a risk assessment tool in adolescent males.

### Supplementary Information

Below is the link to the electronic supplementary material.

Supplementary file1 (DOCX 195 KB)

## Data Availability

The data presented in this article are not readily available because of the potential for personal re-identification of participants in the present sensitive population (incarcerated juveniles). Interested parties should contact Dr. Kent Kiehl (kkiehl@mrn.org) for the data used in this report which may be shared under a data use agreement.

## Footnotes

1 History of traumatic brain injury (TBI), as assessed via self-report from the Rivermead Post Concussion Symptoms Questionnaire (King et al., 1995), was also tested as a potential covariate of interest given previous findings in the literature (Schwartz, 2021). Given its nonsignificant relationship with outcomes of interest, it was not included in the primary multivariate models.

2 See Supplementary Materials, section S1, for additional clinical descriptive data for the sample.

3 A five-year follow-up window was chosen as a midpoint between the year of last release (2011) and the year of last follow-up collection (2021).

4 While interaction effects are most commonly tested solely to model whether the effects of one variable are dependent upon the levels of another, we have chosen to include them in the third block of the multivariate model in order to test whether they confer additional predictive utility beyond the main effects of the underlying variables themselves (Corrado et al., 2004).

5 The rate of felony and violent felony rearrest for this sample of 254 juvenile males with all relevant assessment data (i.e., PCL:YV, IQ, SUD, & TBI) was not statistically different from the larger population of juvenile males at the same facility who did not have sufficient data for analysis of all variables (*n* = 420, see Fig. S1).

6 Given the comparatively low number of non-violent specific felonies, this outcome categorization was not analyzed separately from felonies in general.

7 While the present study is powered well enough to analyze the Hispanic/Latino subset of the sample (*n* = 193) the non-Hispanic/Latino subset is too small to test the same effects (*n* = 59).

8 Due to their lack of relation to general and violent felony rearrest in bivariate analyses performed, age at release and TBI were not included in the primary multivariate models. Lack of effects for these variables may be due to limited range and a comparatively high base-rate, for age and TBI respectively.
