# Supplementary material for: Psychopathy Scores Predict Recidivism in High-risk Youth: A Five-year Follow-up Study
Source: Res Child Adolesc Psychopathol. 2024 Feb 26;52(7):1089–103. doi: 10.1007/s10802-024-01169-x (PMC11217095; doi:10.1007/s10802-024-01169-x)
Supplement: Supplementary file 1 — Supplementary file1 (DOCX 195 KB) [file 10802_2024_1169_MOESM1_ESM.docx]

**Supplementary Materials**

**S.1 Methods**

***S.1.1 Diagnosis of Psychiatric Disorders***

For descriptive purposes, we assessed whether or not participants met criteria for various forms of psychopathology, including anxiety disorders, mood disorders, post-traumatic stress disorder (PTSD), and attention-deficit/hyperactivity disorder (ADHD), we utilized the Kiddie Schedule for Affective Disorders and Schizophrenia (KSADS; Kaufman et al., 1997). Categorization of potential anxiety disorders included obsessive compulsive disorder, generalized anxiety disorder, acute stress disorder, panic disorder (with and without agoraphobia), separation anxiety, phobias (i.e., social phobia and/or specific phobias), agoraphobia, or an anxiety disorder not otherwise specified (NOS). Mood disorders included major depressive disorder (with and without psychotic features), melancholic depression, dysthymia, adjustment disorder with depressed mood, depressive disorder NOS, schizoaffective disorder (depressed and manic types), mania, hypomania, cyclothymia, or bipolar disorder NOS. Based on this criteria, 11 participants met lifetime criteria for any anxiety disorder, 44 participants met criteria for any mood disorder, 33 met criteria for ADHD, and 21 participants met criteria for PTSD.

**S.2 Results**

***S.2.1 Survival Curves of Current Sample Versus Broader Population of Males at the Same Facility***


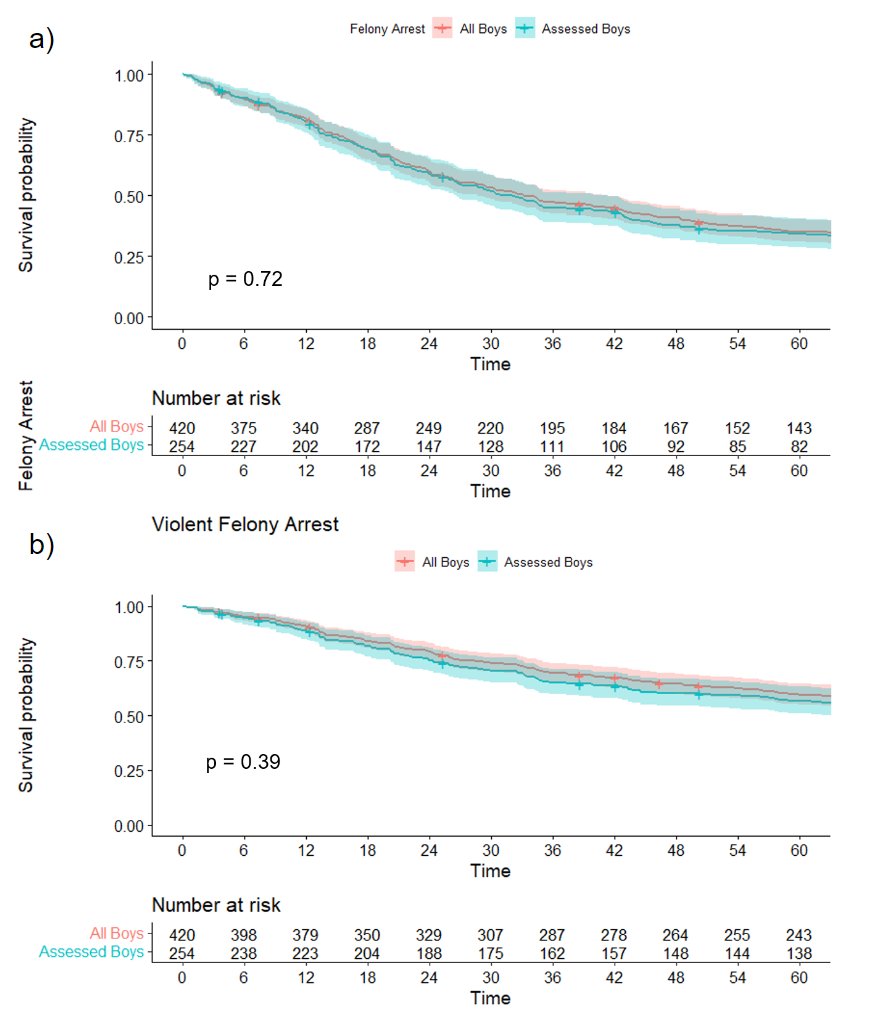


**Figure S1.** Kaplan-Meier curves showing rearrest rates (in months) with 95% confidence intervals and survival risk tables across: a) Felony Rearrest and b) Violent Felony Rearrest, for the present sample with all relevant assessment data (*n* = 254) compared to the larger population of juvenile males at the same facility who consented to our study protocol, yet did not complete relevant assessments (*n* = 420, of which the *n* = 254 is included in). *P*-values from a direct group comparison of survival risk are reported on both Kaplan-Meier curves and suggest no significant difference in rates of rearrest between these two samples.
